# Cross-Reactive Plasmonic Aptasensors for Controlled Substance Identification

**DOI:** 10.3390/s17091935

**Published:** 2017-08-23

**Authors:** Joshua N. Yoho, Brian Geier, Claude C. Grigsby, Joshua A. Hagen, Jorge L. Chávez, Nancy Kelley-Loughnane

**Affiliations:** 1 711th Human Performance Wing, Airman Systems Directorate, Air Force Research Laboratory, Wright-Patterson AFB, Dayton, OH 45433, USA; yohoj1@chem.ufl.edu (J.N.Y.); brian.geier.2@us.af.mil (B.G.); claude.grigsby@us.af.mil (C.C.G.); joshua.hagen.1@us.af.mil (J.A.H.); 2 UES, Inc., 4401 Dayton-Xenia Road, Dayton, OH 45432, USA

**Keywords:** illicit drugs, plasmonic sensor, aptamer, gold nanoparticle, cross-reactive, colorimetric

## Abstract

In this work, we developed an assay to determine if an arbitrary white powder is a controlled substance, given the plasmonic response of aptamer-gold nanoparticle conjugates (Apt-AuNPs). Toward this end, we designed Apt-AuNPs with specific a response to common controlled substances without cross reactivity to chemicals typically used as fillers in street formulations. Plasmonic sensor variation was shown to produce unique data fingerprints for each chemical analyzed, supporting the application of multivariate statistical techniques to annotate unknown samples by chemical similarity. Importantly, the assay takes less than fifteen minutes to run, and requires only a few micrograms of the material, making the proposed assay easily deployable in field operations.

## 1. Introduction

Controlled substance abuse is a great public health concern that affects 5% of the world population aged 15–64 [1], with substance abuse mainly belonging to cannabis, opioid, cocaine, or amphetamine type stimulants. The pre-market rapid identification of controlled substances is critical to help suppress controlled substance accessibility to the public. Ideally, a reliable assay (or set of assays) is necessary to allow law enforcement personnel to quickly identify whether or not a controlled substance is present in the field.

Currently, there are several available tests for drug identification in the field, including the Scott (or cobalt thiocyanate) test for cocaine [2], the modified Duquenois–Levine test for cannabis [3], and Ehrlich Reagents for LSD [4]. These tests have proven to be invaluable to law enforcement operators in helping determining whether or not a powder found at a crime scene is a controlled substance [5,6]. Typically, a substance that is determined by these methods to be an illicit drug is further analyzed in a centralized facility for identity confirmation [6,7]. In general, a critical issue with any test is the probability of false-positive or false negative results, with the latter being a more critical issue in legal cases [8]. Another practical limitation is that screening for multiple controlled substances requires the use of a series of tests, resulting in a process that is impractical, time consuming, and prone to operator errors. Taken together, a simple fieldable test that can reliably determine if a suspicious powder is one of several controlled substances would provide an unmet capability critically needed by law enforcement personnel.

The development of sensing approaches for the detection of drugs of abuse and their metabolites in biofluids is a very active research area, with traditional analytical techniques like HPLC and MS dominating these efforts [9,10,11]. New technologies based on nanomaterials [12] and the combination of nanoparticles and biorecognition elements (BREs) [13] have been reported focusing on the detection of traditional drugs like cocaine and benzocaine. Importantly, in most cases, each assay or sensing platform responds to one illicit drug, which means that the identification of multiple drugs in the field would require the use of a number of assays or sensors in parallel, which could be cumbersome and time-consuming. Recently, electrochemical methods have been reported for the detection of up to three cathinone derivatives on street samples with the use of screen-printed electrochemical sensors [14,15], and up to eleven compounds of the “spice” family with commercially available electrodes [16], showing great potential to develop point-of-use sensors for multiple illicit drugs.

Cross-reactive sensors are proposed here as a means to match the identity of an unknown sample against a large number of illicit drugs in a simple format. In addition to the evident cost- and time-saving feature of this sensing approach, these sensors offer a number of advantages compared to single-analyte assays. These including built-in redundancy, which has been shown to minimize false positive rates [17], the ability to potentially cover any analyte of interest [18], and, by using proper data analysis tools, the proposed sensor outputs can be trained to assign an unknown to a member of the training set [19] by using statistically derived similarity. A simple route to develop cross-reactive sensors utilizesAuNPs plasmonic response to different analytes [20]. Coupling AuNPs with different BREs allows for the tuning of the AuNPs’ response to specific targets of interest. For example, Rotello et al. used small AuNPs (~2 nm), coupled to a series of ligands whose fluorescence was quenched to different degrees by the AuNPs after binding to the analytes of interest. These nanomaterials allowed the design of cross-reactive sensors for cells and tissues [21], proteins, and bacterial biofilms [22]. In a more field-friendly approach, changes in the extinction of larger AuNPs (~15 nm) due to analyte binding can be measured directly by the naked eye without the need for tags in the BRE, as demonstrated by Jayawardena et al., using carbohydrate ligands to identify different lectins [23]. The use of the intrinsic plasmonic signals of the AuNPs simplifies the sensor design since the AuNPs extinction can be measured with simple instrumentation, including smartphone devices, expanding their use to applications in areas with limited resources [24].

Aptamers are one of the most promising BREs due to their ease of synthesis, stability, and chemical versatility [25,26]. For example, it has been demonstrated that aptamers and AuNPs can be combined to create plasmonic cross-reactive sensors (Apt-AuNPs) for proteins and cells [20]. However, small molecular targets, with simpler chemical structures and a low number of functional groups are more challenging targets to identify. Our group has recently demonstrated that Apt-AuNPs can be designed to create cross-reactive sensors for small molecular analytes, allowing for target identification and quantification [27]. In this work, our goal was to design a set of Apt-AuNPs that could respond to different controlled substances to obtain a fingerprint for each chemical that can in turn be used to identify a suspect substance in the field. The intended operational situation involves finding a white powder in a crime scene that needs to be rapidly tested before being submitted to a centralized lab for identity confirmation. Therefore the assay was developed in a field-friendly format, minimizing sample size, simplifying sample treatment, and reducing assay time, and it was tested with commercially available certified standards.

To test the potential application of the proposed sensing platform to the identification of a large number of drugs of abuse, we selected a variety of illicit drugs with diverse chemical structures, including traditional drugs: cocaine, LSD, oxycodone, and morphine; and substances classified under the new psychoactive substances (NPS) category: two synthetic cannabinoids widely used in Europe and the USA [11,28]: 1-pentyl-3-(1-naphthoyl)indole (JWH-018, also referred to as “spice” [29]), and *N*-(1-adamantyl)-1-pentyl-1H-indazole-3-carboxamide (also known as Apinaca or AKB48 [30]); and two synthetic cathinone analogs: methylenedioxypyrovalerone (MDPV) [31], found in street samples in Italy as recently as of 2015 [30], and a newly developed MDPV analog, 4-methyl methcathinone (Mephedrone), which abuse in European countries has recently resulted in a “Critical Review Report” by the World Health Organization Expert Committee in Drug Dependence [32].

## 2. Materials and Methods

### 2.1. Materials

The following chemicals: gold (III) chloride, sodium citrate, HEPES (4-(2-hydroxyethyl)-1-piperazineethanesulfonic acid) sodium salt, magnesium chloride, lactose, sucrose, inositol, caffeine, and diethyl pyrocarbonate (DEPC) were purchased from Sigma-Aldrich (St. Louis, MO, USA), Milli-Q water was filtered with a Q-Pod from Millipore (Darmstadt, Germany), DNA aptamers were purchased from Integrated DNA Technologies (IDT) (Coralville, IA, USA) and purified by standard desalting, methanol was purchased from OmniSolv (Charlotte, NC, USA), 1 mg/mL methanolic certified standards of Apinaca (AKB48), cocaine (COC), 1-pentyl-3-(1-naphthoyl)indole (JWH-018), lysergic acid diethylamide (LSD), 4-methyl methcathinone (4-MMC), morphine (MOR), methylenedioxypyrovalerone (MDPV), and oxycodone (OXY) were purchased from Lipomed (Cambridge, MA, USA). All the controlled substances purchased as 1 mg/mL solutions were listed in the “Exempt Chemical Preparation List” released by the DEA [33].

### 2.2. Aptasensor Preparation

AuNPs were prepared by standard methods. A 500 mL Erlenmeyer flask containing a stirring bar was cleaned with a mixture of 9 mL of concentrated HCl and 3 mL of concentrated HNO_3_. A mixture of 98 mL of Milli-Q water and 2 mL of 50 mM HAuCl_4_ was added, and the flask was covered and sealed with aluminum foil and allowed to boil; at which point 10 mL of 38.8 mM sodium citrate was added to the solution. Heating continued until a deep red color was observed, and continued for 10 min, after which it was left to incubate and stir at room temperature (RT). After cooling, 100 μL of DEPC was added and the solution was placed back on the stir plate at room temperature overnight. The following day the solution was autoclaved and filtered with a Corning filtration system with a CA cellulose membrane of 0.22 μm. The final AuNP concentration was determined with a Cary 300 UV-Bio Spectrophotometer (Varian, Palo Alto, CA, USA), by monitoring the AuNPs’ extinction at 530 nm. The extinction coefficient value of these particles is 2.4 × 10^8^ M^−1^·cm^−1^. DNA was added to AuNPs at a ratio of 90:1 (MN4-AuNPs) and 180:1 (EBA-AuNPs), after which it was left to sit for several hours, followed by an addition of an equivalent volume of 2× assay buffer (20 mM HEPES, 2 mM MgCl_2_) and overnight incubation. This was also done to c-AuNPs, but in this case, no DNA was added in the process. The Apt-AuNPs were ready to use after the overnight incubation in buffer.

### 2.3. Plasmonic Assay Design and Optimization

Assay optimization was performed as follows: MeOH and/or a methanolic solution of the analyte being tested, (10 μL) was added to 90 μL of Apt-AuNPs, and after 30 s, 8 μL of salt was added to the solution. Several wells, on a 96 well Costar Black plate, were run at the same time, using a range of salt concentrations to promote a strong response to the analytes of interest with minimal signal from MeOH (the blank). A Synergy HT plate-reader from Biotek (North Shoreline, WA, USA) was used to monitor the changes in AuNP extinction at 650 nm (aggregated AuNPs), and 530 nm (dispersed AuNPs) after the salt addition. Measurements were taken every minute for 7 min. The aggregation degree (defined as the ratio of aggregated/dispersed AuNPs = E_650_/E_530_) was monitored over time. Kinetic data was plotted through Graph Pad (La Jolla, CA, USA). The assay was then run using six replicates of the same target, followed by the addition of the optimized salt concentration. Each replicate was used to develop the average and standard deviation, from which it was determined that the five most consistent data sets were used. In all tests, digital images were taken 2 min after salt addition in the optimized assay, using a Canon Rebel T3i camera. The assay was designed to use a methanolic solution of the powder of interest. This was done to allow the dissolution of the hydrochloride salt of certain substances (like cocaine) and their free bases. Also, typical filler substances like sucrose, lactose, and inositol, do not dissolve in this solvent, minimizing the effect of these substances in the assay response. In order to test the response of the assay to these species, controlled substances were diluted from a 1 mg/mL methanolic solution, to 0.5 mg/mL with assay buffer, and the fillers substances were prepared as a 1 mg/mL in assay buffer and diluted to 0.5 mg/mL with MeOH. The assay was run as explained above, and the NaCl concentration needed was optimized for this system. RGB values were established using the “RGB Measure” function on ImageJ (Wayne Rasband, NIH, Bethesda, ML, USA).

### 2.4. Colorimetric Response Characterized by Dynamic Light Scattering (DLS) and Transmission Electron Microscopy (TEM)

DLS measurements were performed in a ZetasizerNano instrument (Malven Instruments, Westborough, MA, USA) utilized in back scatter mode (173° detection angle) with the temperature set at 20.0 ± 0.1 °C. The target (10 μL) was added to the Apt-AuNPs, and the size analysis was started after 30 s. Finally, similarly to what is done in the optimized assay, 8 μL of NaCl was added to promote Apt-AuNPs aggregation. However, the concentration in these studies was weaker than the salt concentration used in the optimized assay to prevent fast aggregation, which would confound the data analysis due to the formation of large aggregates during the measurement. TEM samples were prepared by mixing 90 μL of AuNPs, 10 μL of target and 8 μL of salt; the salt concentration was lower than the one used in the assay, to prevent over aggregation. After about one minute, around 3 μL of solution was removed and placed onto a 200 mesh copper grid with a formvar carbon film (Electron Microscopy Sciences, Hatfield, PA, USA), after which it was allowed to dry out overnight before images were taken with a Hitachi H-7600 (Hitachi Ltd., Tokyo, Japan).

### 2.5. Data Clustering and Classification

An illicit drug training set was designed for the plasmonic sensors developed in this work. The three sensors were challenged with eight different controlled substances, each tested in five replicates, resulting in a 3 × 8 × 5 matrix. Before using the software XLSTAT (New York City, NY, USA) to analyze the data for the different analytes, the values were adjusted by removing the background. This was done by determining the average value of MeOH and subtracting these values from the respective control samples. The adjusted values were then run through Discriminant Analysis (DA), which performs a supervised cluster analysis. DA implemented algorithms that classified the data into separate clusters. These results were further verified for accuracy and reliability by implementing a leave-one-out method of unlabeled sampling. In this approach, one member of the data set is used for validation, and the rest as the training set. The result is given as a table indicating the failure rate. Finally, a Gaussian test was run as well, which utilizes an unsupervised classification approach, to investigate the intrinsic data separation.

## 3. Results

**Figure 1:**
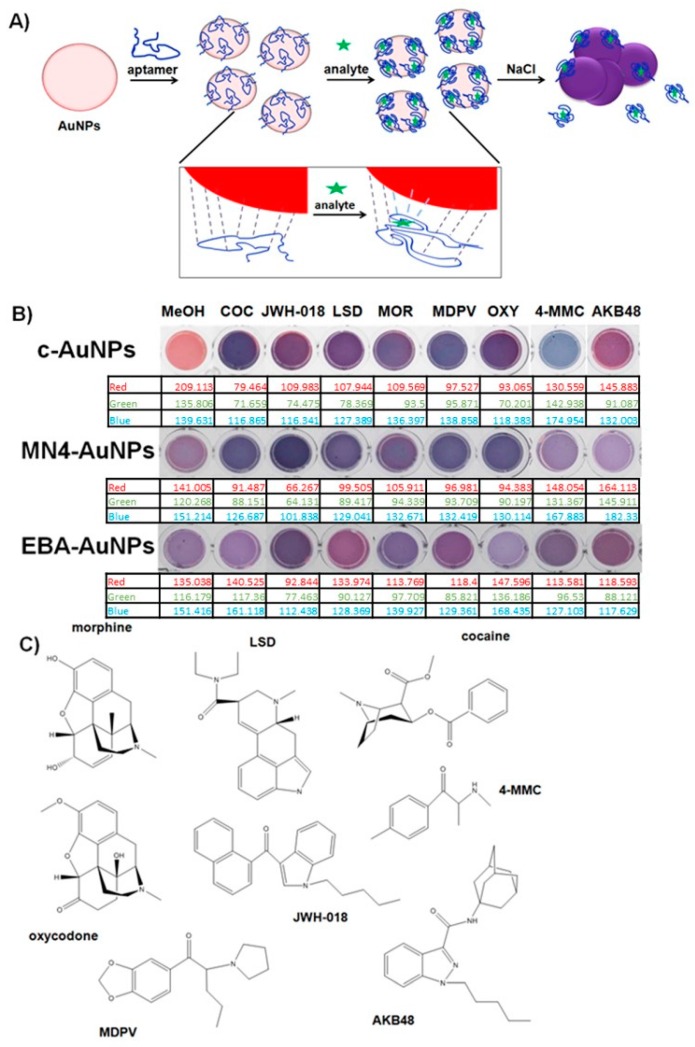
Rationale for controlled substance assay design. (**A**) Schematic representation of the Apt-AuNP conjugates-based plasmonic assay. The inset shows the proposed mechanism of cross-reactive response, in which the interactions between the aptamer, analyte and AuNPs are unique depending on the analyte, resulting in different responses; (**B**) Colorimetric and RGB response obtained with the Apt-AuNPs after exposure to different controlled substances; (**C**) Chemical structures of controlled substances used.

Plasmonic sensors based on AuNPs typically function based on the disruption of particle stability due to the binding of an analyte of interest [25,26,34]. Selectivity is typically achieved by using a BRE coupled to the AuNPs surface and engineering the BRE binding event to control AuNPs aggregation, resulting in a change in color in the AuNPs suspension when the target of interest is detected. In this work, we aimed to control AuNP aggregation by adsorbing aptamers on their surface, and affect AuNPs stability as a consequence of the binding event, as shown in Figure 1A. Citrate stabilized-AuNPs (c-AuNPs) can interact with a number of species due to their affinity for different functional groups that can coordinate with the metal surface. For instance, citric acid, a simple molecule composed of alcohol groups, coordinate to the AuNPs surface with a well-defined geometry [35]. Nucleotides are known to displace these molecular ions from the AuNPs surface through interactions between the metal atoms and the nitrogen groups [36]. We hypothesized that the affinity of the AuNPs for different chemical moieties can provide a tool to design a sensor platform that can respond to different molecules. The controlled substances studied here (see Figure 1C for chemical structures) have different chemical functionalities, including varying numbers of alcohol and amine groups, which could result in the AuNPs interacting with these chemicals to different degrees, potentially resulting in different plasmonic responses. It is important to note that these small molecular targets with simple structures and low numbers of functional groups are more difficult to discriminate with cross-reactive sensors, as compared to proteins or cellular targets, which have a high density of chemical moieties.

**Figure 2:**
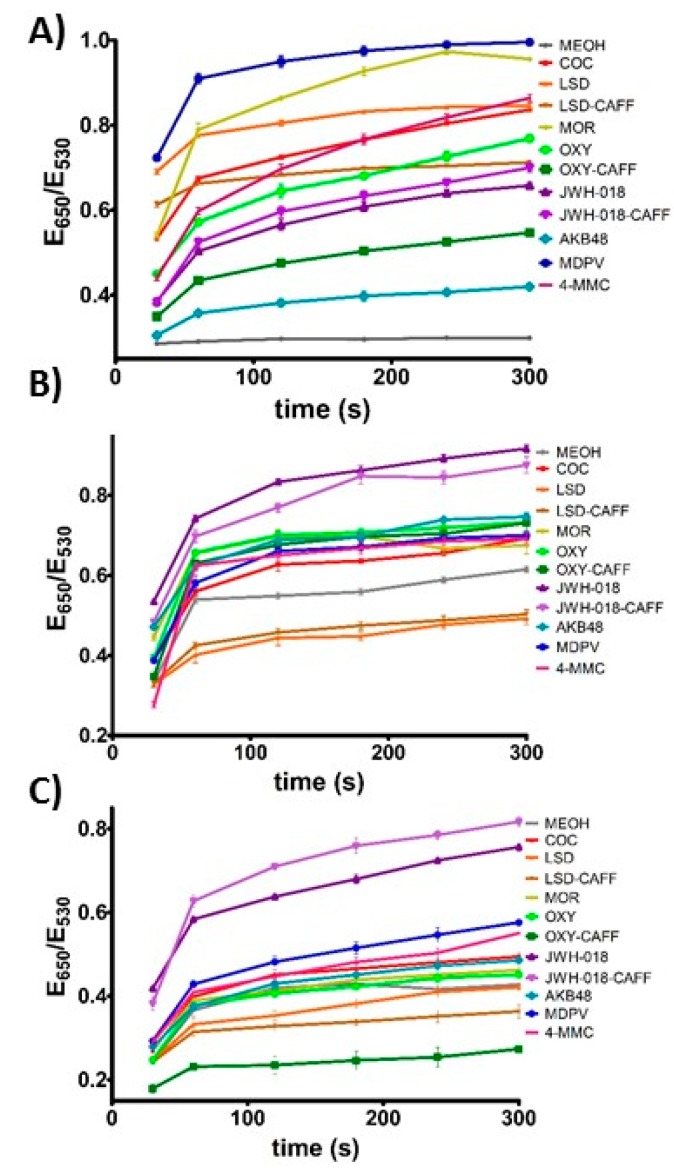
Controlled substance assay kinetic response. The analytes dissolved in methanol (500 µg/mL), added to the Apt-AuNPs and after 30 s particle aggregation was promoted by addition of a final NaCl concentration of: (**A**) c-AuNPs (40 mM); (**B**) EBA-AuNPs (120 mM) and (**C**) MN4-AuNPs (80 mM).

To test the ability of c-AuNPs to provide plasmonic responses to different controlled substances, they were exposed to different methanolic solutions of these compounds and mixtures of illicit drugs with caffeine, a typical masking agent used in street formulations, followed by the addition of NaCl to promote c-AuNP aggregation. As shown in Figure 1B, exposing c-AuNPs to the analytes tested here resulted in a variety of plasmonic responses, providing an easy-to-read colorimetric output. Supplementary Figure S1 shows preliminary data used to determine the optimized salt concentration and incubation time with salt needed to obtain a low background response with the solvent and a large and unique response for each of the analytes of interest. It can be observed that most of the plasmonic response to the analytes is obtained at earlier times and that the data plateaus after approximately 2 min. It is important to consider that this effect is partially controlled by the amount of NaCl in the assay, which is carefully selected during the assay optimization. As shown in Figure 1B, analysis of the RGB values of the pictures for the different samples demonstrate the ability to differentiate the response of the assay to different analytes. However, a more precise and simple way to differentiate this response can be obtained by monitoring the degree of aggregation of the Apt-AuNPs by monitoring the extinction of the AuNPs at 530 nm (dispersed AuNPs), and 650 nm (aggregated AuNPs). Figure 2A shows quantification of the AuNPs aggregation degree, given as the ratio of the extinction of aggregated (E_650_) and dispersed (E_530_) AuNPs, with larger values corresponding to severe aggregation, and lower values indicating a minimal effect on particle stability. The data showed that the interactions between the c-AuNPs and the different analytes tested resulted in different degrees of the plasmonic response, suggesting that this system could be used to create cross-reactive sensors for controlled substances (Figure 2A). Importantly, none of the typical filler substances used in the street samples affected the c-AuNPs stability (Figure S2).

In order to provide better discrimination for analyte identification and increase the robustness of the sensors, we created two Apt-AuNPs utilizing aptamers that were observed in preliminary experiments to provide consistent and reliable cross-reactive responses to the controlled substances of interest. Two aptamers were used to create the plasmonic sensors, a widely studied cocaine-binding aptamer with a three-way junction structure (MN4) [37], and an aptamer that was selected to bind 17-β-estradiol (EBA) [38], with a simple structure composed of long stretches of paired bases, resulting in two plasmonic sensors, MN4-AuNPs and EBA-AuNPs, respectively. We hypothesized that the significantly different structural features of these two aptamers would allow for different DNA-analyte interactions to occur on the AuNPs surface, providing cross-reactive responses for different targets. As shown in Figure 2B, the MN4-AuNPs responded to controlled substances with the response intensity depending on the analyte used. A digital image of the colorimetric response is shown in Figure 1B, showing a different color pattern than the c-AuNPs. Importantly, no response to the filler chemicals was observed (Figure S2). We have observed in our previous work that the affinity of EBA is greatly broadened when used in the Apt-AuNP sensor format [25], providing a tool to detect multiple targets with different affinities. As expected, different levels of response were observed when the EBA-AuNPs were exposed to the different controlled substances, as shown in the digital image in Figure 1B. Quantification of the response to the different analytes confirmed that each analyte promoted a different degree of aggregation, as shown in Figure 2C.

**Figure 3:**
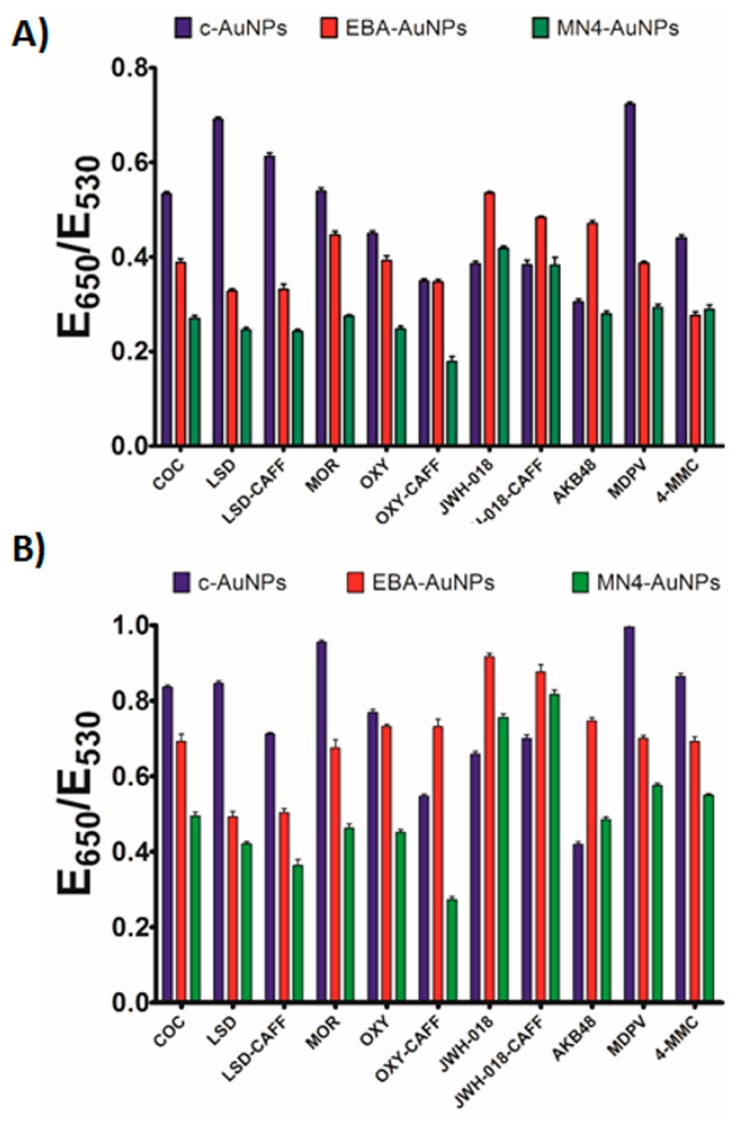
Controlled substances plasmonic fingerprint. The response of the Apt-AuNPs is presented grouped by analyte tested after incubation with NaCl to promote color development for: (**A**) 30 s; (**B**) 5 min.

A comparison of the responses observed with the c-AuNPs, MN4-AuNPs, and EBA-AuNPs, confirmed that the use of different aptamers provided a tool to tune the assay response to controlled substances, see Figure 3 for analyte fingerprints. The data plot in Figure 3A was obtained by utilizing the response of each Apt-AuNPs under optimized conditions after thirty seconds incubation time with salt, while Figure 3B was the response after five-minute incubation time with salt. It is clearly observed that the combined response of the three plasmonic sensors allowed for the differentiation of the controlled substances fingerprint, which can be utilized to match the fingerprint of an unknown to one of the eight substances of interest for law enforcement duties. Moreover, this response is easily observed by the naked eye, and can be coupled to data analysis in a mobile device, as we have previously shown [39]. As expected, based on the kinetic data shown in Figure 2, different incubation times after salt addition, promote responses at different rates, offering one more parameter to control the sensors output and optimize analyte identification (Figure 3A,B).

To test the applicability of this sensing platform to real samples, we turned our attention to mixtures of illicit drugs and caffeine, a commonly used masking agent. Caffeine showed a minimal effect on the assay response by itself. However, when caffeine was mixed with the controlled substances studied here, the effect on the assay response depended on the specific illicit drug chemical structure and the Apt-AuNP used. The response to JWH-018 mixed with caffeine was very similar to pure JWH-018 in all cases. A similar case was observed with LSD and the LSD-caffeine mixture, except in the case of the c-AuNPs, where the presence of caffeine seemed to mask the response by approx. 25%. The EBA response to oxycodone was unaffected by the presence of caffeine, but a significant decrease in the signal response was observed with the c-AuNPs and MN4-AuNPs (approx. 50% signal inhibition). The different effects observed by the presence of caffeine with the different compounds and Apt-AuNPs is probably due to the variety of possible interactions between caffeine, the specific drug used, and the Apt-AuNPs surface. Ideally, one would prefer that the presence of this masking agent did not affect the assay response, however, since what makes this sensing platform attractive is its ability to provide a different signal when different interactions between the target and the sensor are encountered, this effect can be used to classify the pure and mixed samples individually, as will be demonstrated in the next section.

**Figure 4:**
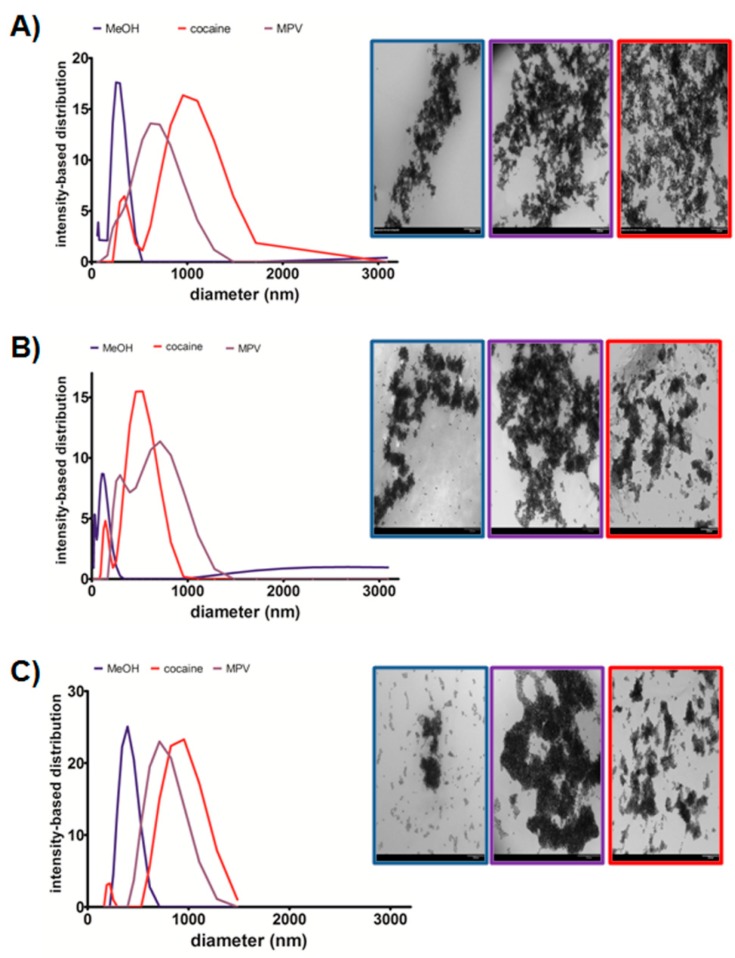
DLS and TEM characterization. Left: Apt-AuNP size distribution as determined by DLS after NaCl addition, Right: color-coded TEM images after NaCl addition of: (**A**) c-AuNPs, (**B**) MN4-AuNPs, and (**C**) EBA-AuNPs, after exposure to methanol (blue trace), cocaine (red trace) and MDPV (purple trace). Scale bar, 200 nm.

To confirm that the plasmonic response observed in the assay was due to analyte-binding triggered AuNP aggregation, we studied the mechanism of the color response by DLS. The NaCl concentration used in the DLS experiments was lower than in the assay, to keep a slow aggregation pace and facilitate the tracking of the AuNPs size changes during the analyte addition and salt-induced aggregation steps. The AuNPs were exposed to methanol, cocaine, or MDPV, and incubated for 15 min, followed by the NaCl addition. The size distribution plots in Figure 4 demonstrated that the AuNPs exposed to the solvent underwent a mild aggregation as the ionic strength of the media was increased; while exposure to cocaine and MDPV resulted in serve aggregation, which shifted the AuNP average size to the micrometer range. We propose that the analyte-induced aggregation results from a combination of interactions between the analytes, the DNA, and the AuNPs surface. To confirm this hypothesis, we characterized the color generation process by TEM. Figure 4 shows micrographs of Apt-AuNPs after the addition of analyte and incubation with NaCl. We focused our attention to two analytes, MDPV and cocaine, which resulted in a strong response with the different Apt-AuNPs and the blank as a comparison. To avoid overaggregation induced by the drying process, the salt concentration for TEM characterization was typically 50% less than what was used in the optimized assay. The images showed that, in all cases, the AuNPs agglomerate severely after NaCl addition in the presence of the targets but show minor aggregation in the presence of the solvent (blank), as shown in Figure 4. Figure S3 shows the size distribution before and after NaCl addition. Therefore, both TEM and DLS demonstrated that the color change observed in the assay was due to the presence of the target that affected the AuNPs stability, resulting in different degrees of aggregation depending on the analyte being sensed and the DNA used.

**Figure 5:**
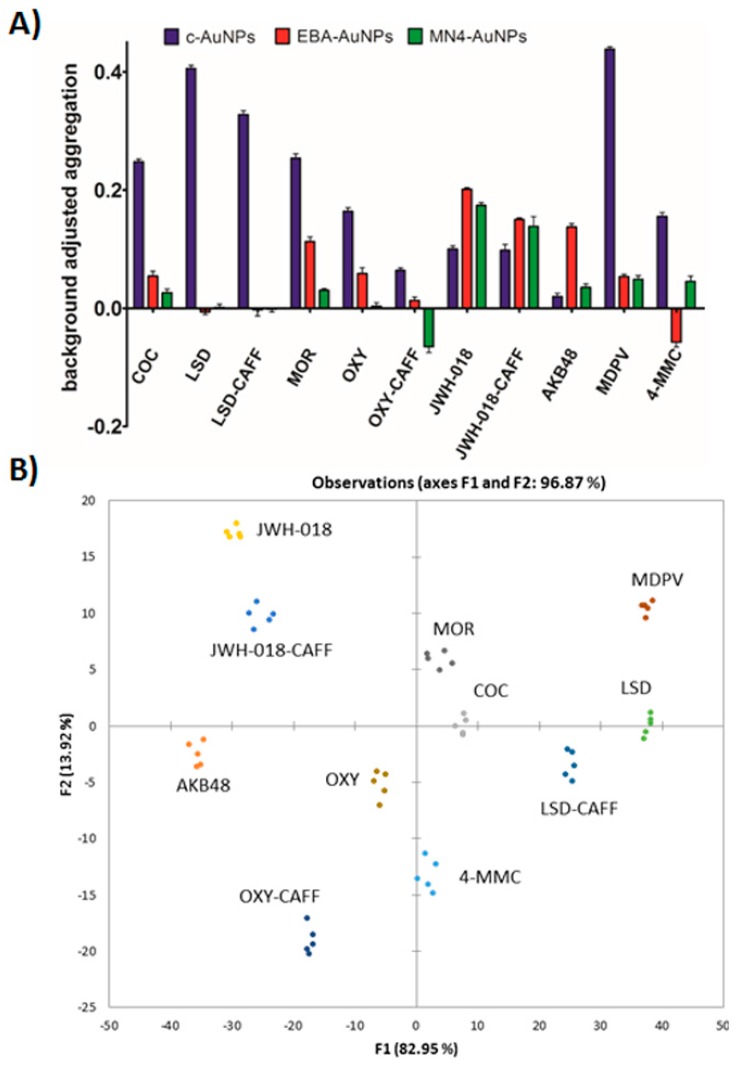
Controlled substance classification. (**A**) Background corrected aggregation data (30 s incubation with NaCl) used as an input in discriminant analysis; (**B**) Clustering plot based on the first two canonical factors.

The data shown in Figure 1 and Figure 2 suggested that the plasmonic response of the c-AuNPs, MN4-AuNPs, and EBA-AuNPs could be combined to provide a means to match the identity of an unknown to one of eight controlled substances. We have previously demonstrated that principal components analysis (PCA) of Apt-AuNPs cross-reactive sensors data could be used to identify and quantify different targets [27]. We applied the same rationale to the plasmonic fingerprint obtained in this work with the aim of identifying whether the sample of interest is one of the eight illicit drugs studied. Our initial analysis demonstrated that discriminant analysis (DA) provided more compact clusters and better inter-cluster separation, as compared to the PCA [40]. Therefore, the optimized assay response was analyzed by DA using the software XLSTAT after testing the system with commercially available certified standards. The data set consisted of five replicates of eight analytes with the response of the three plasmonic sensors (resulting in a 5 × 8 × 3 matrix). Figure S4 shows that the analysis of the raw data resulted in sub-optimal clustering with some overlap between the 4-MMC and cocaine clusters, which would prevent efficient analyte identification. Importantly, as shown in Figure 5A, the background-corrected data, the difference between the response to each analyte and the response obtained under the same assay conditions to the blank (methanol), provided a better input for our analysis [19].

Discriminant analysis of the corrected data of the three plasmonic sensors after thirty seconds incubation post-salt addition resulted in three canonical factors (see Table S1 for input values for DA and Table S2 for output values). Visualization of the first two canonical factors, accounting for 82.95% and 13.92% of the variation, respectively, resulted in distinct clusters with no overlap, suggesting that unambiguous analyte identification was possible, as shown in Figure 5B. Importantly, oxycodone and morphine, with very closely related chemical structures were successfully resolved, showing the potential of these Apt-AuNP sensors as cross-reactive sensors. To gauge predictive strength, we performed the leave-one-out cross-validation analysis with the data set, obtaining no classification errors [41], see Table S3 in the Supplementary Information. Additionally, prolonged incubation time was associated with higher EBA-AuNPs signal variance, but c-AuNPs signal variance remained stable across incubation times tested, respectively. Statistical significance was assessed by fitting a single factor analysis of variance model to within class standard deviations for each signal separately. Geometrically, the within class standard deviation is simply the ellipsoids axis aligned spread. As shown in Figure S5, as incubation time increases, the EBA-AuNPs spread, or variance within a class also increases (F(2,42) = 12.95; *p* < 0.01). Furthermore, a controlled incubation time is necessary to minimize measurement variance, and hence, improve elliptical boundaries between classes.

These results confirm the potential of the approach developed here for designing plasmonic cross-reactive sensors based on aptamers and AuNPs. By using a simple and fast assay, the identity of an unknown can be matched unambiguously to one of six controlled substances.

## 4. Conclusions

In this work, a colorimetric assay based on the plasmonic response of Apt-AuNPs to different controlled substances was designed to test the identity of a powder found in the field. The affinity of the AuNPs was tuned to controlled substances by using aptamers that recognized the different functional groups present in drugs of abuse. Clearly identifiable fingerprints were obtained for each drug tested, utilizing the AuNPs aggregation degree as the assay readout. The potential of this approach to identify an unknown sample as a drug of abuse was demonstrated by successfully clustering the data in an unsupervised fashion. Importantly, the assay is very simple and requires only four steps: dissolving the powder in methanol, adding the sample to the Apt-AuNP suspension, adding salt and allowing color development. The three tests can be run in parallel (for instance, in contiguous wells in a 96 well plate) with a total assay time of less than 15 min. We acknowledge that we have demonstrated that the current system works with simple mixtures, but complex mixtures containing multiple active ingredients are a much more challenging scenario. We believe that the discrimination power of the current assay could be significantly improved by using a larger library of aptamer sequences to diversify the chemistry involved in the recognition step. We envision that these types of sensors could greatly benefit from sequences selected with tuned cross-reactivity. This could be accomplished by incorporating the combination of a set of desired targets as the selection “analyte”, and evolve the selected binders to recognize a family of targets. We believe that this approach shows great potential to design an easy-to-use assay for controlled substances with on-the-spot analysis. Importantly, this sensor platform could be tuned to respond to other types of compounds by choosing the right aptamer combination.

## Supplementary Materials

The following are available online at http://www.mdpi.com/1424-8220/17/9/1935/s1, Figure S1: Assay Optimization, Figure S2: Comparison of Assay Response to Controlled Substances and Fillers, Figure S3: DLS data showing particle sizes before and after addition of salt, Figure S4: DA classification of non-corrected sensor data, Figure S5: Assay Scatterplot Matrix. Table S1: Input data for discriminant analysis, Table S2: Output data for discriminant analysis, Table S3: Leave-one-out cross validation results obtained with analysis performed on the dataset shown in Table S1.
