# Supplementary material for: Cross-Reactive Plasmonic Aptasensors for Controlled Substance Identification
Source: Sensors (Basel). 2017 Aug 23;17(9):1935. doi: 10.3390/s17091935 (PMC5620944; doi:10.3390/s17091935)
Supplement: Supplementary file 1 [file sensors-17-01935-s001.pdf]

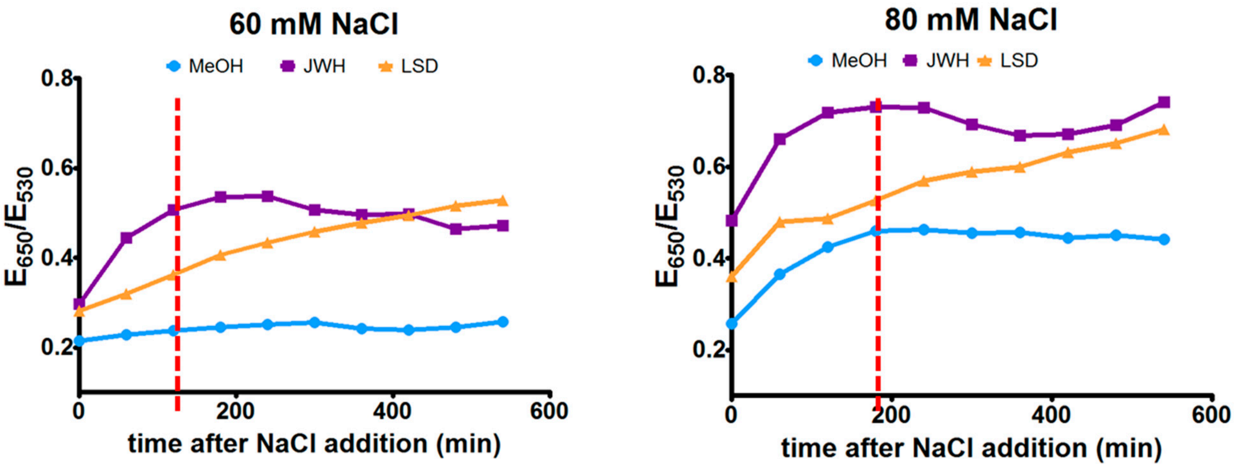

Figure S1. Assay Optimization. Initial trial to identify the optimal salt concentration and incubation time with salt to obtain different assay responses to the analytes of interest using the MN4-AuNPs. The red lines indicate the proposed optimal test time for each salt concentration tested.

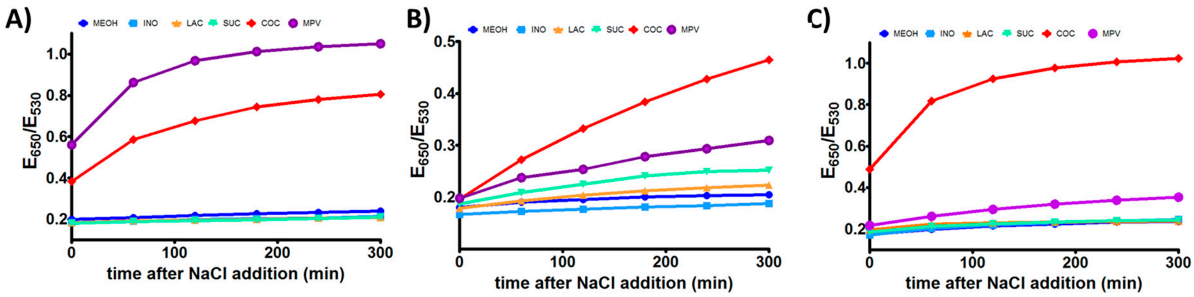

Figure S2. Comparison of Assay Response to Controlled Substances and Fillers. Analytes were dissolved in a 1:1 mixture of methanol:buffer to a concentration of 0.5 mg/mL. Apt-AuNPs were mixed with the analytes and incubated for 30 seconds before NaCl addition. Apt-AuNPs extinction was measured immediately after salt addition: (**A**) c-AuNPs, (**B**) MN4-AuNPs and (**C**) EBA-AuNPs.

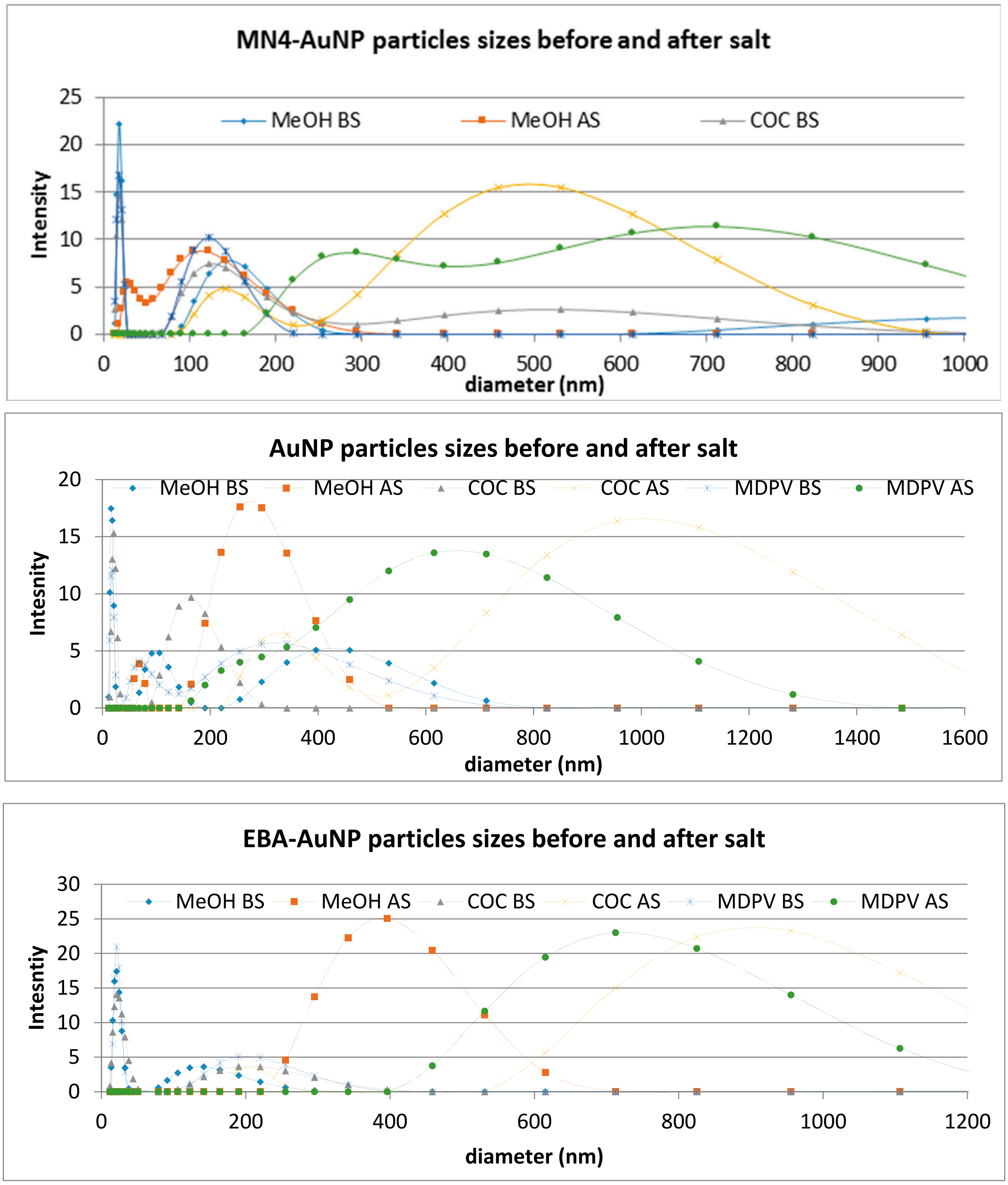

Figure S3. Dynamic light scattering data showing particle sizes before and after addition of salt. Each sensor shows the trend that the addition of salt causes the particles to destabilize and aggregate. The target also has an influence on stability, for methanol typically has the smallest size, showing the least reaction with the AuNP.

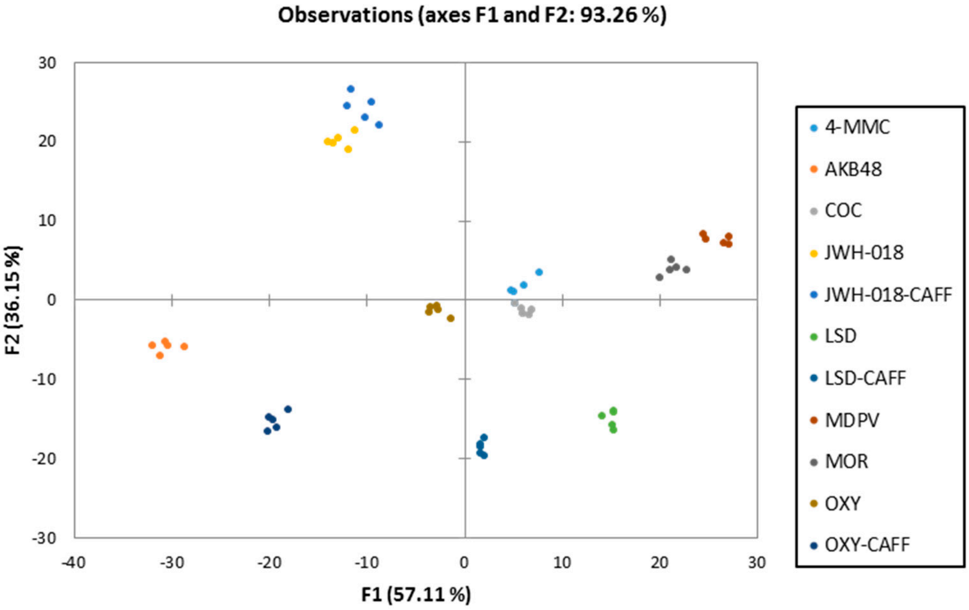

Figure S4. Differential analysis classification of non-corrected sensor data, data was recorded after three minutes incubation time with salt.

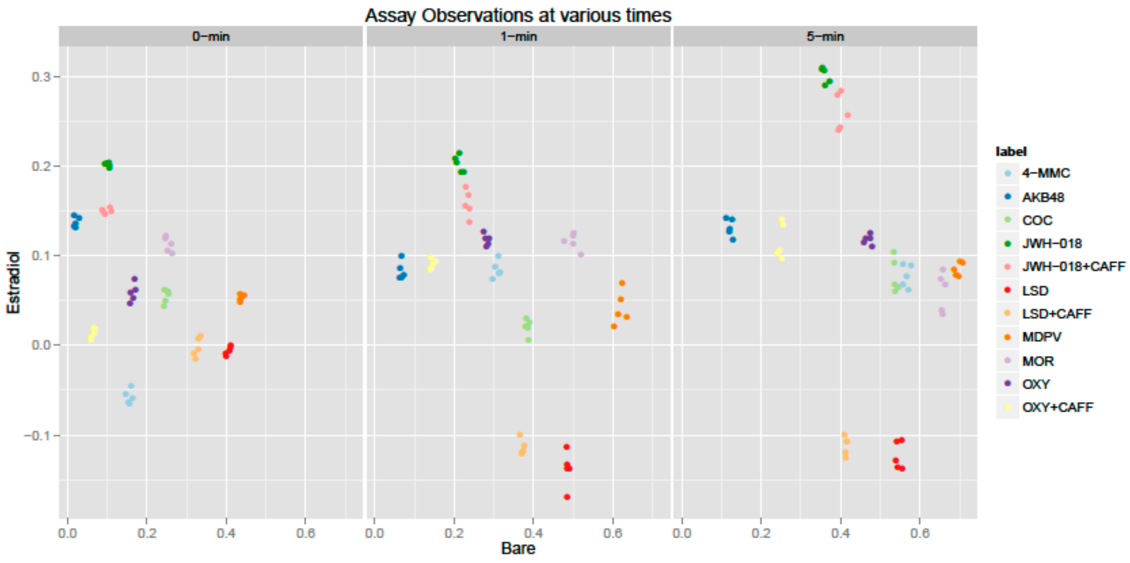

(**a**)

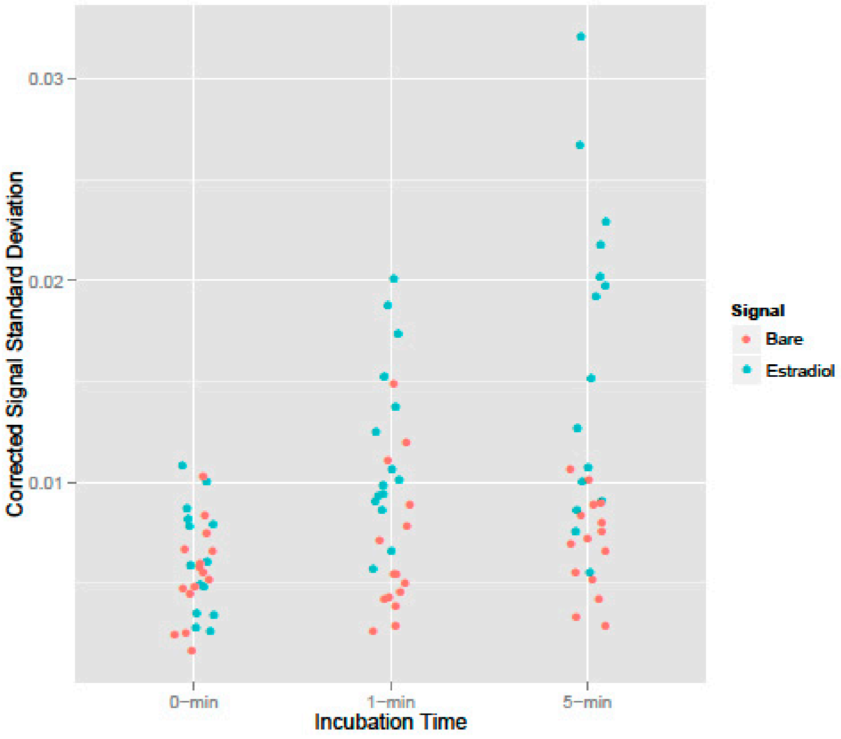

(**b**)

Figure S5. (**a**) Assay Scatterplot Matrix. C-AuNPs (Bare) and EBA-AuNPs (Estradiol) Signals are plotted against each other showing the separation between various chemical classes at either 0, 1, or 5 minute incubation times. (**b**) Variance comparison between c-AuNPs (Bare) and EBA-AuNPs (Estradiol) at 0, 1, or 5 minute incubation. Note increased Estradiol measurement variance at longer incubation time. Variance is computed from 5 replicates for each chemical class (e.g., OXY, OXY+CAFF, etc.) and incubation time.

Table S1. Input data for discriminant analysis of assay response training set (30 s).

	Bare	Estradiol	MN4
COC	0.242747	0.043034	0.032422
COC	0.244034	0.062167	0.018335
COC	0.253313	0.057461	0.02521
COC	0.252413	0.05968	0.032008
COC	0.246154	0.050114	0.023525
JWH−018	0.092773	0.201931	0.178824
JWH−018	0.103827	0.204169	0.179728
JWH−018	0.095444	0.202929	0.16916
JWH−018	0.104125	0.200342	0.170512
JWH−018	0.104125	0.196993	0.171582
LSD	0.398833	−0.00912	−0.00807
LSD	0.409389	−0.00352	0.002378
LSD	0.407837	−0.00623	0.002414
LSD	0.399593	−0.01259	0.005095
LSD	0.411216	0.000197	0.004428
MOR	0.251978	0.105866	0.026715
MOR	0.260599	0.113454	0.034492
MOR	0.246414	0.118502	0.028738
MOR	0.247573	0.12216	0.029224
MOR	0.262727	0.102793	0.031923
MDPV	0.434748	0.048838	0.058796
MDPV	0.435382	0.05123	0.040697
MDPV	0.439548	0.053891	0.046526
MDPV	0.43521	0.057297	0.047991
MDPV	0.445161	0.056135	0.049713
OXY	0.171144	0.061445	0.00789
OXY	0.164933	0.052371	0.002808
OXY	0.158335	0.058637	0.010613
OXY	0.168007	0.07338	−0.00257
OXY	0.157347	0.046786	−0.00257
JWH−018−CAFF	0.093932	0.146455	0.162545
JWH−018−CAFF	0.109971	0.148952	0.133489
JWH−018−CAFF	0.089943	0.149995	0.125958
JWH−018−CAFF	0.087418	0.150911	0.147592
JWH−018−CAFF	0.107195	0.153682	0.121859
LSD−CAFF	0.33574	0.010434	−0.00777
LSD−CAFF	0.329774	0.006722	0.004414
LSD−CAFF	0.322931	−0.01503	−0.00314
LSD−CAFF	0.330476	−0.00432	−0.0023
LSD−CAFF	0.318852	−0.0098	0.001099
OXY−CAFF	0.065197	0.019133	−0.04701
OXY−CAFF	0.05945	0.00945	−0.06856
OXY−CAFF	0.066207	0.012819	−0.07173
OXY−CAFF	0.058462	0.005348	−0.0687
OXY−CAFF	0.069613	0.018486	−0.06903
AKB48	0.028672	0.142253	0.041106
AKB48	0.019799	0.130954	0.03008
AKB48	0.019084	0.135689	0.037095
AKB48	0.016252	0.132409	0.028259
AKB48	0.015758	0.144836	0.039653
4−MMC	0.152492	−0.06345	0.043322

4−MMC	0.159755	−0.04522	0.053286
4−MMC	0.163302	−0.05933	0.055216
4−MMC	0.146143	−0.05433	0.042341
4−MMC	0.155352	−0.06471	0.032487

Table S2. Output data for discriminant analysis of assay response training set shown in Table S1.

Observation	Prior	Posterior	F1	F2	F3
Obs1	COC	COC	7.608	−0.830	−0.719
Obs2	COC	COC	6.368	−0.028	2.424
Obs3	COC	COC	8.160	0.472	1.423
Obs4	COC	COC	7.765	1.122	0.907
Obs5	COC	COC	7.651	−0.661	0.861
Obs6	JWH−018	JWH−018	−30.800	17.212	−3.765
Obs7	JWH−018	JWH−018	−29.242	18.003	−3.521
Obs8	JWH−018	JWH−018	−30.363	16.734	−2.649
Obs9	JWH−018	JWH−018	−28.786	17.003	−2.904
Obs10	JWH−018	JWH−018	−28.519	16.773	−3.308
Obs11	LSD	LSD	37.078	−1.175	0.870
Obs12	LSD	LSD	38.182	0.588	0.421
Obs13	LSD	LSD	38.160	0.270	0.160
Obs14	LSD	LSD	37.354	−0.510	−0.779
Obs15	LSD	LSD	38.143	1.163	0.559
Obs16	MOR	MOR	3.921	4.957	5.495
Obs17	MOR	MOR	4.581	6.619	5.470
Obs18	MOR	MOR	1.972	6.000	6.323
Obs19	MOR	MOR	1.847	6.425	6.609
Obs20	MOR	MOR	5.827	5.557	4.827
Obs21	MDPV	MDPV	37.297	10.645	−0.468
Obs22	MDPV	MDPV	37.381	9.592	1.611
Obs23	MDPV	MDPV	37.762	10.452	1.298
Obs24	MDPV	MDPV	36.777	10.665	1.391
Obs25	MDPV	MDPV	38.434	11.151	1.239
Obs26	OXY	OXY	−5.022	−4.280	2.504
Obs27	OXY	OXY	−5.203	−5.771	2.152
Obs28	OXY	OXY	−6.847	−4.945	1.814
Obs29	OXY	OXY	−6.403	−4.085	4.586
Obs30	OXY	OXY	−5.889	−7.027	2.118
Obs31	JWH−018−CAFF	JWH−018−CAFF	−25.855	11.007	−6.939
Obs32	JWH−018−CAFF	JWH−018−CAFF	−23.229	9.901	−3.526
Obs33	JWH−018−CAFF	JWH−018−CAFF	−26.414	8.512	−2.916
Obs34	JWH−018−CAFF	JWH−018−CAFF	−27.107	10.033	−5.094
Obs35	JWH−018−CAFF	JWH−018−CAFF	−23.944	9.368	−1.951
Obs36	LSD−CAFF	LSD−CAFF	25.453	−2.331	1.746
Obs37	LSD−CAFF	LSD−CAFF	24.693	−2.076	0.092
Obs38	LSD−CAFF	LSD−CAFF	25.487	−4.938	−1.126
Obs39	LSD−CAFF	LSD−CAFF	25.786	−3.539	−0.176
Obs40	LSD−CAFF	LSD−CAFF	24.364	−4.345	−1.155
Obs41	OXY−CAFF	OXY−CAFF	−17.758	−17.102	3.087
Obs42	OXY−CAFF	OXY−CAFF	−17.651	−19.811	4.381
Obs43	OXY−CAFF	OXY−CAFF	−16.827	−19.412	5.089
Obs44	OXY−CAFF	OXY−CAFF	−17.466	−20.245	4.024
Obs45	OXY−CAFF	OXY−CAFF	−16.784	−18.538	5.352
Obs46	AKB48	AKB48	−34.635	−1.190	4.350

Obs47	AKB48	AKB48	−34.995	−3.437	4.380
Obs48	AKB48	AKB48	−35.571	−2.532	4.064
Obs49	AKB48	AKB48	−35.659	−3.602	4.650
Obs50	AKB48	AKB48	−36.882	−1.666	4.561
Obs51	4–MMC	4–MMC	2.018	−14.065	−12.328
Obs52	4–MMC	4–MMC	1.559	−11.335	−11.663
Obs53	4–MMC	4–MMC	3.271	−12.323	−13.052
Obs54	4–MMC	4–MMC	0.266	−13.599	−11.509
Obs55	4–MMC	4–MMC	2.685	−14.826	−11.287

Table S3. Leave-one-out cross validation results obtained with analysis performed on the dataset shown in Table S1.

From \ To	4-MMC	AKB48	COC	JWH-018	JWH-018-CAFF	LSD	LSD-CAFF	MDPV	MOR	OXY	OXY-CAFF	Total	Correct %
4-MMC	5	0	0	0	0	0	0	0	0	0	0	5	100.00%
AKB48	0	5	0	0	0	0	0	0	0	0	0	5	100.00%
COC	0	0	5	0	0	0	0	0	0	0	0	5	100.00%
JWH-018	0	0	0	5	0	0	0	0	0	0	0	5	100.00%
JWH-018-CAFF	0	0	0	0	5	0	0	0	0	0	0	5	100.00%
LSD	0	0	0	0	0	5	0	0	0	0	0	5	100.00%
LSD-CAFF	0	0	0	0	0	0	5	0	0	0	0	5	100.00%
MDPV	0	0	0	0	0	0	0	5	0	0	0	5	100.00%
MOR	0	0	0	0	0	0	0	0	5	0	0	5	100.00%
OXY	0	0	0	0	0	0	0	0	0	5	0	5	100.00%
OXY-CAFF	0	0	0	0	0	0	0	0	0	0	5	5	100.00%
Total	5	5	5	5	5	5	5	5	5	5	5	55	100.00%
